# Supplementary material for: The breakome of BRCA1 and BRCA2 pathway mutation carriers reveals early processes in breast oncogenesis
Source: Cell Death Dis. 2025 Dec 5;16(1):891. doi: 10.1038/s41419-025-08235-2 (PMC12717187; doi:10.1038/s41419-025-08235-2)
Supplement: Supplementary file 1 — Supplementary data [file 41419_2025_8235_MOESM1_ESM.zip › Supplementary material/Supplementary figure legends.docx]

**Figure Legends**

**Supplementary Fig 1:** The Breakome of Normal and *BRCA* Mutated Primary Cells can be Distinguished

A. Integrative genomics viewer (IGV) software was used to visualize the genome-wide DSB pattern of primary breast cells in BigWig format, and demonstrates similarities and differences between the breakome of normal primary breast cells and *BRCA*-mutated ‘high risk’ primary breast cells. B. Correlation plot (Pearson) highlights similarities and differences between breakome of samples across binned whole genome. C. Correlation-based similarity index represents the mean correlation between each group (whithin *BRCA*^mut^, within Normal) and the correlation between the two groups. Correlation values were averaged for each group and plotted as shown. Statistics were calculated using Wilcoxon test.

**Supplementary Fig 2:** Genes more broken in *BRCA* mutated cells are transcriptionally associatedA. Breakome vs. expression analysis demonstrates a higher expression distribution for genes that are highly broken, more prominently so in *BRCA* mutated samples. Genes in each break category were plotted based on their expression levels. Statistics were measured using Wilcoxon test. B. OncoKB tumor suppressors and proto-oncogenes are more susceptible to breakage in *BRCA* mutated samples. Highly or lowly broken were defined based on the differential breakage of *BRCA* mutated cells. Statistics were measured using Wilcoxon test. C. Methylation loss genes and D. Methylation loss promoters demonstrate higher break density in *BRCA* mutated cells compared to average risk controls at a higher level than observed in methylation gain genes/promoters. Statistics were measured using one-tailed Wilcoxon test. E. *BRCA* mutated samples have higher break density in genes which are associated with methylation loss in breast DCIS tumors. Statistics were measured using one-tailed Wilcoxon test.

**Supplementary Fig 3:** Characterization of DNA double strand breaks across breast cell lines

A. Distribution of breaks across genomic sites shows break enrichment across genes and especially at the 5’ UTR and promoter regions. Bars represent the normalized break count enrichment per selected site for each cell line. ‘Expected’ depicts the representation of each site in the genes across the genome, making it the point for comparison. B. Breaks are enriched in genes that are early replicating. Time-of-Replication analysis depicts break density across TOR categories from late replicating genes (left) to early replicating genes (right). P-value was calculated using Kruskal-Wallis similarity test. C. Breaks are enriched in short genes. Gene-length analysis depicts break density across gene length categories from short genes (left) to long genes (right). P-value was calculated using Kruskal-Wallis similarity test.

**Supplementary Fig 4:** DNA Double Strand Break Shift of High Risk Primary Cells Corresponds to Breast Cancer Mutation Frequency and HR Repair

A. SNV, B. Deletion or C. Insertion coding gene mutation frequency is shown for differential genes. SNV and Deletion coding gene mutation frequency is higher in *BRCA* mutated Differential genes’ breakome compared to Normal Differential genes’ breakome. Statistics were measured using Wilcoxon test. D. Insertion gene mutation frequency is shown in relationship to normal primary cell gene breakome and *BRCA*-mutated cell gene breakome. Breakome vs. mutation analysis demonstrates a higher mutation frequency for genes that are highly broken in BRCA^mut^ samples. Statistics were measured using Wilcoxon test and Pearson correlation across all of the categories was measured. E. Insertion gene mutation frequency shows no significant advantage in *BRCA* mutated Differential genes’ breakome compared to Normal Differential genes’ breakome. Statistics were measured using Wilcoxon test. F. (same as D) Normal and *BRCA*-mutated Breakome vs. mutation analysis was similarly preformed for rearrangement mutation density. Statistics were measured using Wilcoxon test and Pearson correlation across all of the categories was measured. G. Gene mutation frequency is shown relative to BRCA^mut^ Breakome and Expression categories (Breakome^high^Expression^high^ or Breakome^low^Expression^high^), to show that expression alone is not a determiner of mutation. Statistics were measured using Wilcoxon test.

**Supplementary Fig 5:** Samples with germline *PALB2* mutations considered high risk demonstrate a partially similar phenotype to *BRCA* mutated samples.

A. Principal component analysis of the breakome across the genome, binned in 500kbp bins. The analysis demonstrates that most *PALB2*-mutated samples exhibit different patterns than average risk samples. B. Heatmap of normalized break density at differentially broken genes (P < 5%). Rows (bins) and columns (samples) are clustered according to their spearman correlation. C. Venn diagram shows more genes in common between differentially highly broken genes in *BRCA* mutated samples and *PALB2* mutated samples compared to lowly broken genes. D. Volcano plot shows gene break shifts between *PALB2* mutated (positive log2FC) and normal cells (negative log2FC). Horizontal dashed line represents threshold of P <0.05. Vertical dashed line represents Log2 fold change =1. Genes were marked and labeled if they met the threshold and considered an Oncogene/Tumor Suppressor in OncoKB database. E. Breakome vs. expression analysis demonstrates a higher expression distribution for genes that are highly broken in *PALB2* mutated samples. Genes in each break category were plotted based on their expression levels. F. Methylation loss genes and G. Methylation loss promoters demonstrate higher break density in *PALB2* mutated cells compared to average risk controls at a higher level than observed in methylation gain genes/promoters. Statistics were measured using one-tailed Wilcoxon test. H. Correlation plot highlights similarities and differences between breakome of samples across binned whole genome. I. Heatmap (Spearman hierarchical clustering) of the ‘differentially broken genes’ distinguishing *PALB2* mutated from normal primary cells with inclusion of breast cancer cell line samples demonstrates a partially similar behavior of gene breaks between high-risk *PALB2*^mut^ primary cells and breast cancer cell lines in genes obtained from DESeq output. J. Homologous recombination marker RAD51 enrichment in MCF7 breast cancer associated with gene breakome in *PALB2* mutated samples. Breakome vs. repair analysis demonstrates a higher breast cancer-associated RAD51 binding for genes that are highly broken in *PALB2* mutated cells. Statistics were measured using Wilcoxon test. K. SNV, Insertion or Deletion gene mutation frequency is shown in relationship to PALB2-mutated cell gene breakome. Breakome vs. mutation analysis demonstrates a higher mutation frequency for genes that are highly broken. Statistics were measured using Wilcoxon test and Pearson correlation across all of the categories was measured.
