# Supplementary figures and images for: The breakome of BRCA1 and BRCA2 pathway mutation carriers reveals early processes in breast oncogenesis
Source: Cell Death Dis. 2025 Dec 5;16(1):891. doi: 10.1038/s41419-025-08235-2 (PMC12717187; doi:10.1038/s41419-025-08235-2)

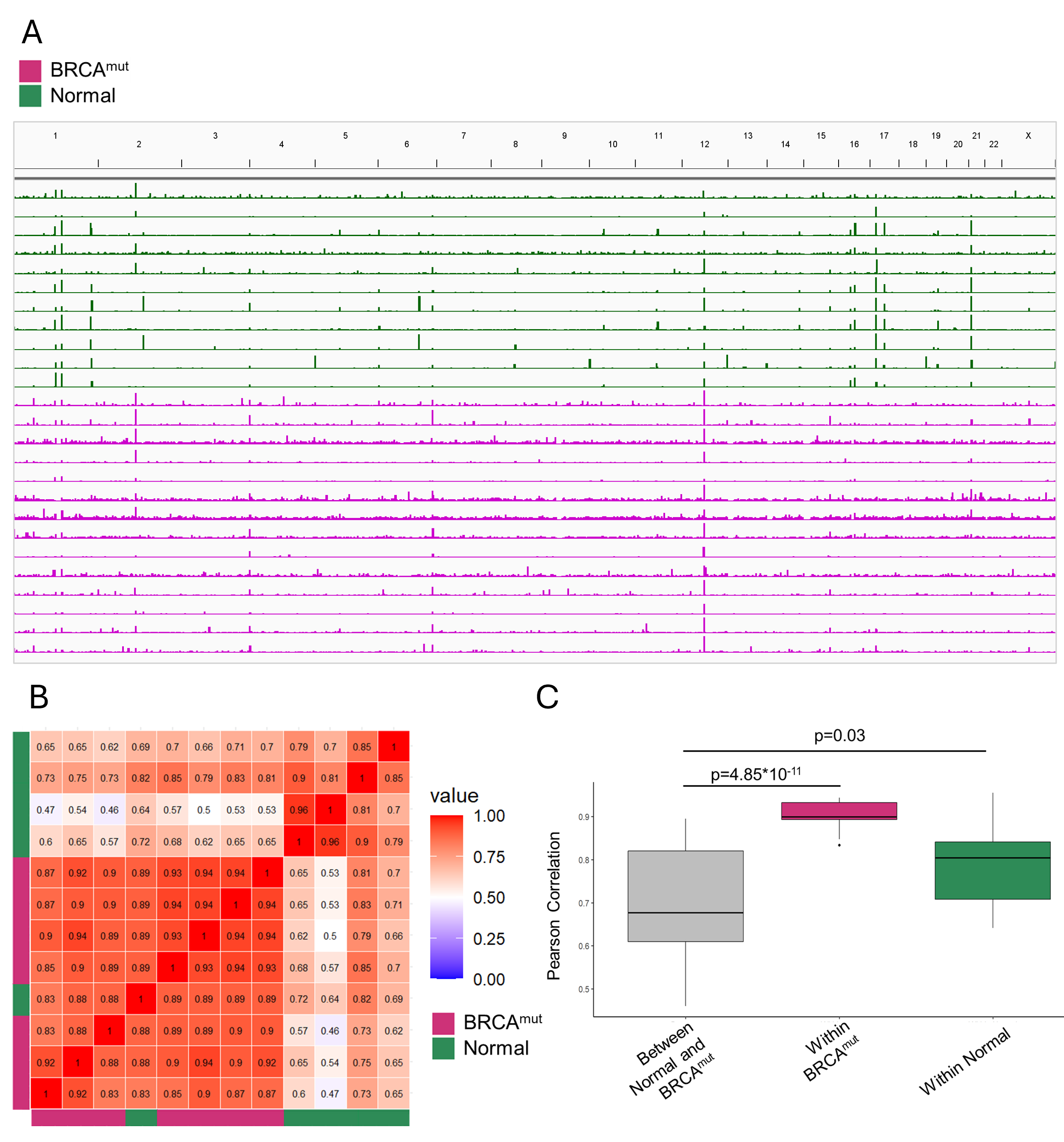

Supplement: Supplementary file 1 — Supplementary data [file 41419_2025_8235_MOESM1_ESM.zip › Supplementary material/Supplementary Figure 1.tif]

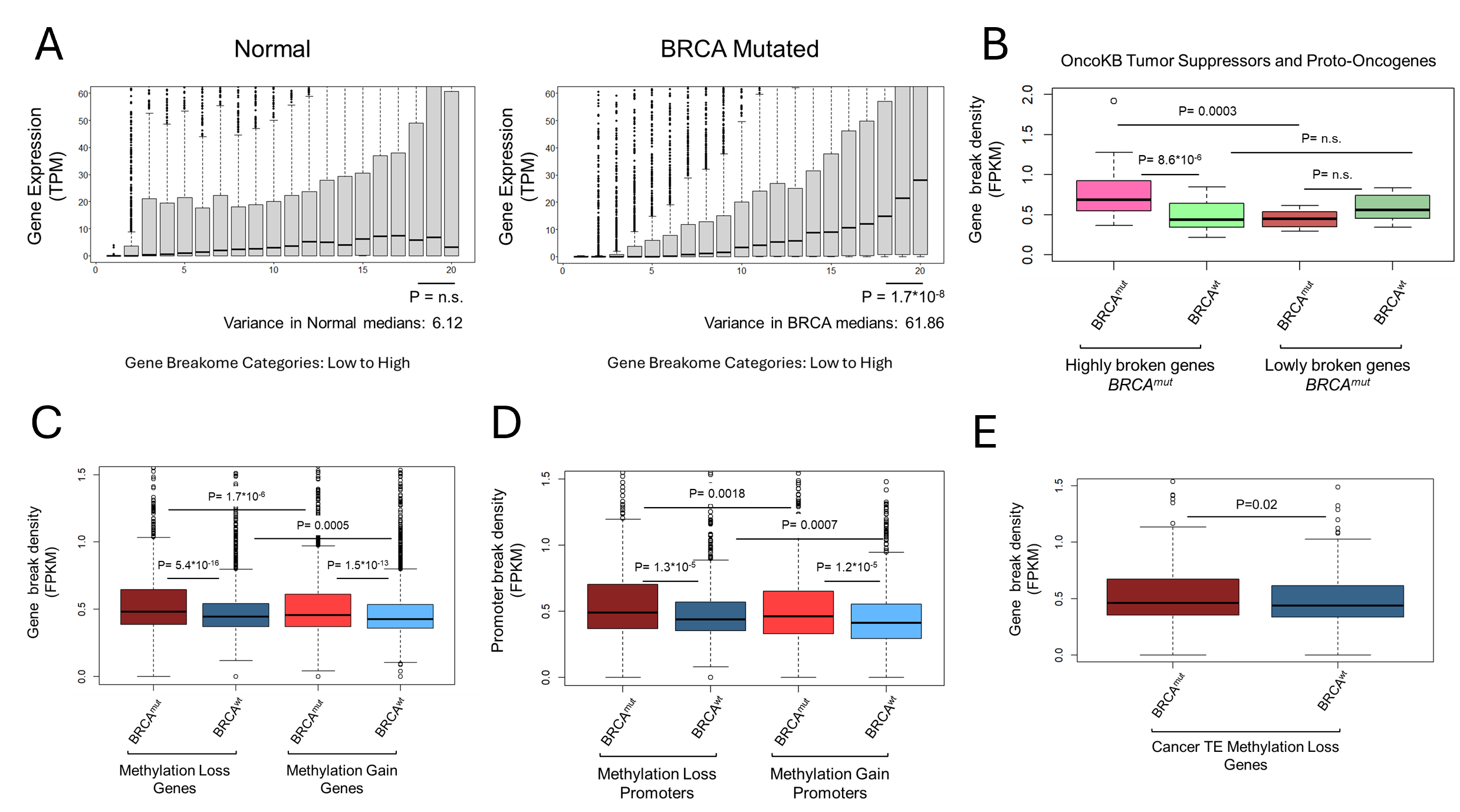

Supplement: Supplementary file 1 — Supplementary data [file 41419_2025_8235_MOESM1_ESM.zip › Supplementary material/Supplementary Figure 2.tif]

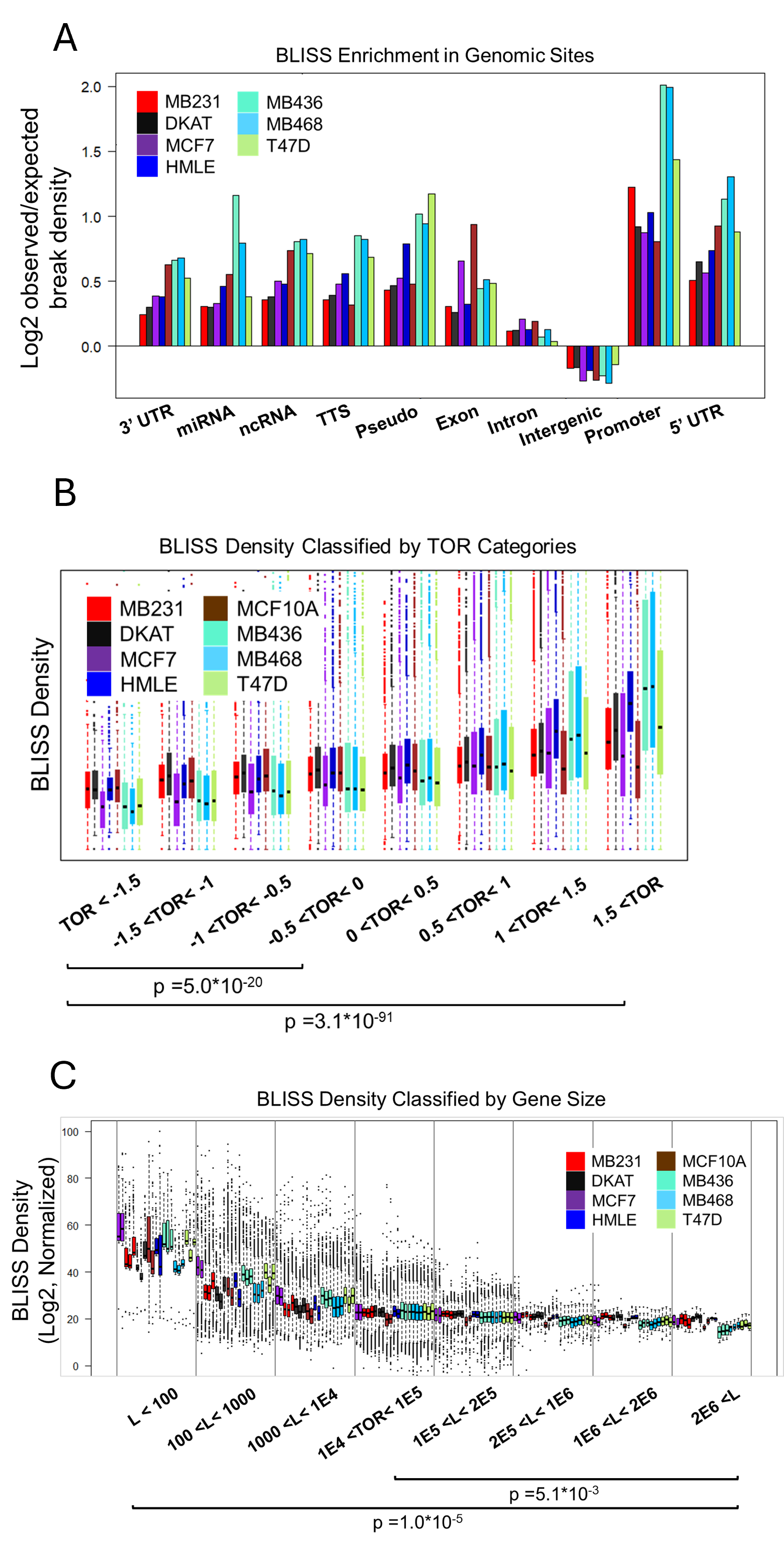

Supplement: Supplementary file 1 — Supplementary data [file 41419_2025_8235_MOESM1_ESM.zip › Supplementary material/Supplementary Figure 3.tif]

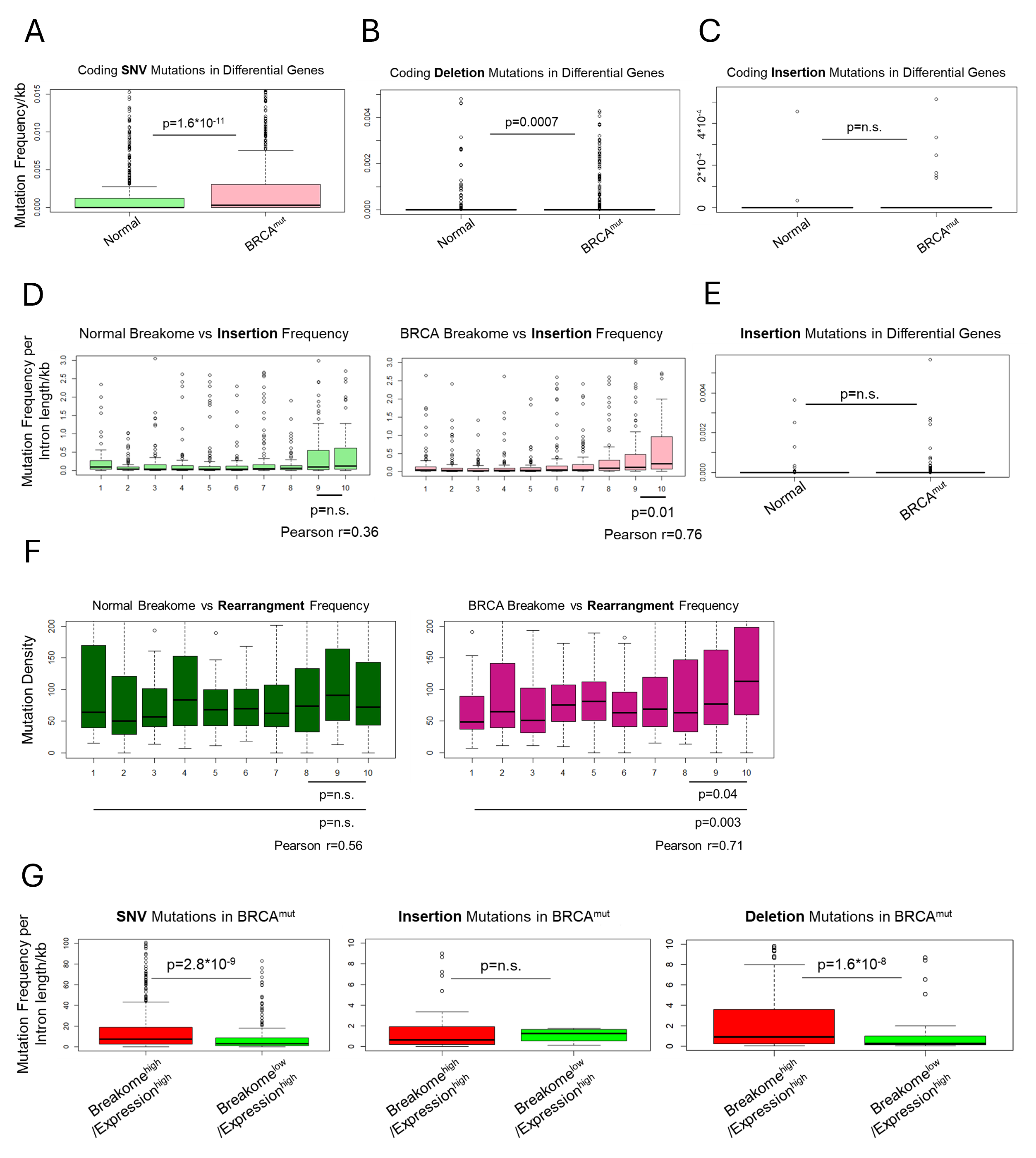

Supplement: Supplementary file 1 — Supplementary data [file 41419_2025_8235_MOESM1_ESM.zip › Supplementary material/Supplementary Figure 4.tif]

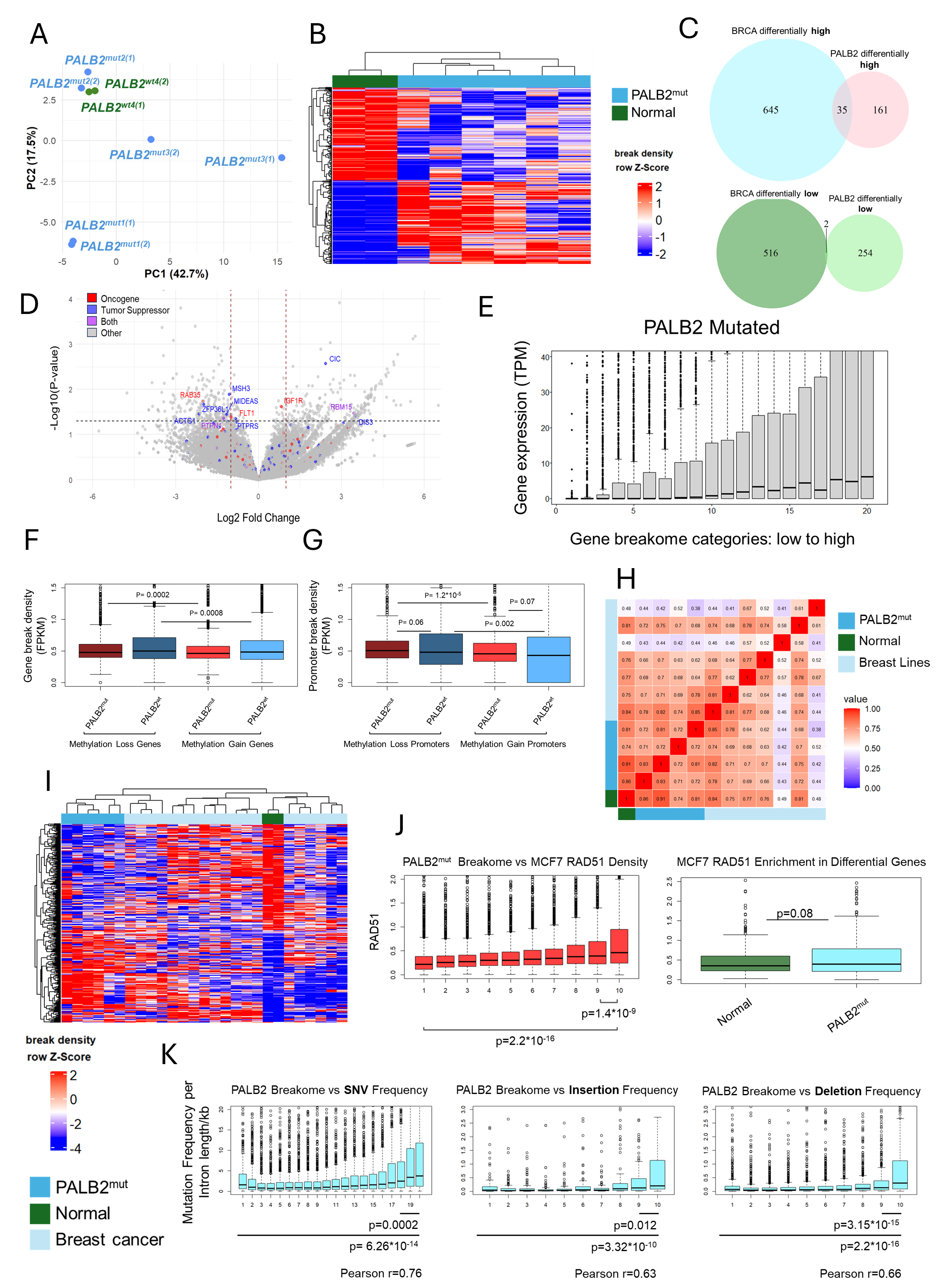

Supplement: Supplementary file 1 — Supplementary data [file 41419_2025_8235_MOESM1_ESM.zip › Supplementary material/Supplementary Figure 5.tif]
